# The Role of NAT10-Mediated ac4C Modification in Osteoblast Function and Bone Formation: Insights from Integrative Bioinformatics and Experimental Validation

**DOI:** 10.33549/physiolres.935656

**Published:** 2026-04-01

**Authors:** Yuchen TANG, Qiufu WANG, Wei DONG, Guanyin JIANG, Miao LEI, Xin HU, Yongle WU, Wei JIANG, Jie HAO, Zhenming HU

**Affiliations:** 1 Department of Orthopedics, University-Town Hospital of Chongqing Medical University, Chongqing, China; 2 Department of Spinal Surgery, Chongqing Orthopedic Hospital of Traditional Chinese Medicine, Chongqing, China; 3 Department of Orthopedics, Nanchuan Hospital of Chongqing Medical University, Chongqing, China; 4 Department of Orthopedics, The First Affiliated Hospital of Chongqing Medical University, Chongqing, China

**Keywords:** ac4C, *NAT10*, Osteoporosis, Bone formation, Osteoblast

## Abstract

Recent evidence has established a significant link between N4–acetylcytidine (ac4C) mRNA modification, mediated by N–Acetyltransferase 10 (*NAT10*), and bone metabolism. Nonetheless, the precise role and regulatory targets of *NAT10*, along with its associated ac4C modification in human bone formation, remain inadequately characterized. This study employed bioinformatics analysis of transcriptomic datasets from primary osteoblasts of individuals with high versus low bone mineral density (BMD), alongside a curated set of ac4C-modified genes, to identify key differentially expressed genes (DEGs) regulated by this pathway within an osteogenic context. Overall, eleven key *NAT10*/ac4C-associated DEGs linked to BMD status were identified: *CFD*, *CTSF*, *DCXR*, *FADS1*, *GOLIM4*, *IMPA2*, *MLEC*, *NCLN*, *NT5DC2*, *PTGFRN*, and *VASP*. Notably, *FADS1*, *NT5DC2*, and *PTGFRN* emerged as crucial ac4C-modified genes across three machine learning models. Furthermore, the tri-gene signature (*FADS1*/*NT5DC2*/*PTGFRN*) showed excellent diagnostic performance in distinguishing different BMD statuses. In vitro validation using MC3T3-E1 osteoblastic cells revealed that the knockdown of *NAT10* via lentiviral delivery markedly impaired cell proliferation and osteogenic differentiation. This impairment was evidenced by the results of the CCK-8 proliferation assay, alkaline phosphatase staining, and Alizarin Red staining. Additionally, qRT–PCR analysis demonstrated a significant downregulation of *FADS1* and *NT5DC2* expression subsequent to *NAT10* knockdown. These findings underscore the role of *NAT10*-mediated ac4C modification as a pivotal regulator of osteoblast activity and gene expression programs associated with BMD. This research offers novel insights into the regulation of bone metabolism and proposes potential diagnostic markers and therapeutic targets for osteoporosis.

## Introduction

Osteoporosis (OP), characterized by diminished bone mass and compromised bone microarchitecture, is emerging as a significant public health issue worldwide [1]. Statistical evidence suggests that the incidence of OP is progressively increasing among women over the age of 55 and men over the age of 65 [2,3]. Furthermore, the prevalence of OP is further exacerbated by the global trend of an aging population [4]. The etiology of OP is multifaceted, with an imbalance in bone metabolism being a principal factor [5,6]. Specifically, OP arises from a disruption in the equilibrium between bone formation and bone resorption [7]. Moreover, current therapeutic approaches for OP predominantly target these two areas: the inhibition of bone resorption and the promotion of bone formation [7]. However, the majority of existing treatments primarily focus on inhibiting bone resorption, such as denosumab [8], with relatively few drugs available that enhance bone formation. This disproportionate focus in treatment strategies may limit the overall efficacy of OP management. Consequently, a comprehensive investigation into the regulatory mechanisms of osteoblasts is essential and anticipated to yield innovative insights and methodologies for the development of new pharmacological agents targeting bone formation.

N4-acetylcytidine (ac4C) modification represents a recently identified form of mRNA chemical modification that has gained substantial recognition for its significance in various biological processes. Initially, ac4C modifications were predominantly linked to tRNA and rRNA. However, in 2018, Arango *et al.* reported the presence of ac4C modifications in mRNA in the journal *Cell*, highlighting their critical role in mRNA stability and the regulation of translation [9]. Subsequent research identified N-Acetyltransferase 10 (*NAT10*) as the sole enzyme known to catalyze ac4C modification on mRNA [9]. Furthermore, a growing body of evidence suggests that *NAT10* and *NAT10*-mediated ac4C modification are pivotal in the pathogenesis of numerous diseases and represent potential therapeutic targets [10–12]. For instance, *NAT10* regulates the ac4C modification of *HK2* in gastric cancer, thereby promoting glycolytic metabolism and tumor progression [13]. Additionally, studies have demonstrated that *NAT10* enhances the translation efficiency of *VEGFA* mRNA by facilitating ac4C modification, which in turn mediates central sensitization [14]. Despite these advancements, the mechanisms by which NAT10 regulates osteoblast-specific transcriptional programs in relation to human bone density variation remain unclear. This study seeks to address this gap in knowledge. Therefore, there is increasing scholarly interest in the role, regulation, and mechanisms of *NAT10* and *NAT10*-mediated ac4C modification in bone metabolism.

Recent studies have elucidated the significant regulatory role of *NAT10* and its associated ac4C modification in bone metabolism [11,15–20]. Specifically, in the context of bone resorption, numerous investigations have reported a positive influence of *NAT10* and its ac4C modification on osteoclastogenesis [15,18]. For instance, *NAT10* facilitates osteoclast generation in inflammatory bone loss by ac4C modification of Fos mRNA, which in turn accelerates osteoclast differentiation through the upregulation of the MAPK signaling pathway and activation of AP-1 (c-Fos/c-Jun) transcription [15]. Additionally, *NAT10* enhances the translation efficiency of *NFATC1*, thereby promoting osteoclast maturation and contributing to increased bone loss in postmenopausal OP [18]. In the context of bone formation, a growing body of evidence suggests that *NAT10* and its associated ac4C modification exert a beneficial effect on osteoblast differentiation [16,17,19,20]. For instance, *NAT10* facilitates the osteogenic differentiation of bone marrow mesenchymal stem cells (BMSCs) by ac4C-modifying *RUNX2* mRNA, which in turn enhances the stability and protein expression of *RUNX2* mRNA, thereby augmenting the osteogenic potential of BMSCs [17]. Similarly, under orthodontic stress, *NAT10* enhances the osteogenic differentiation of periodontal ligament stem cells by modulating the ac4C level of *BMP2* [19]. Furthermore, Xiao *et al.* demonstrated that the Mijiao formula, a traditional herbal remedy that influences *NAT10*-mediated *RUNX2* mRNA ac4C modification, promotes BMSC osteogenic differentiation and ameliorates OP in ovariectomized rats [16]. Although the number of studies is currently limited, the aforementioned evidence indicates that *NAT10* and its mediated ac4C modification may represent a promising therapeutic target for enhancing bone formation. Consequently, a systematic investigation into the role of *NAT10*-mediated ac4C modification in osteoblast differentiation and bone formation, especially in relation to variations in human bone mineral density (BMD), is essential for enhancing our comprehension of bone metabolism regulation and for the development of innovative therapeutic strategies.

Building on the previously discussed context, this study aims to integrate bioinformatic analysis with in vitro experimental validation to identify key *NAT10*/ac4C-regulated differentially expressed genes in osteoblasts that are associated with variations in BMD. The research will examine the functions, pathways, and predictive potential of these genes in relation to BMD differences. Furthermore, the study intends to identify crucial ac4C-modified genes using machine learning models and to develop a risk prediction model for low BMD. Additionally, the study will preliminarily evaluate the effects of *NAT10* knockdown on osteoblast proliferation and differentiation using the MC3T3-E1 cell model and will validate the expression changes in the identified key *NAT10*/ac4C target genes following the knockdown.

## Methods

### Data availability

The data, encompassing two transcription profiles (GSE156508 [21] and GSE157322 [22]) of primary osteoblasts from patients exhibiting varying BMD levels (low or high), along with their associated clinical information, were procured from the NCBI Gene Expression Omnibus (GEO) database [23] (accessible at https://www.ncbi.nlm.nih.gov/geo/). The datasets were retrieved in MINiML format. Specifically, the GSE156508 dataset was derived from platform GPL16686, while the GSE157322 dataset was based on platform GPL15207. Moreover, the GSE156508 dataset included 12 patients, with 6 individuals characterized by low BMD (undergoing hip replacement due to osteoporotic fracture) and 6 individuals with high BMD (undergoing hip replacement due to severe osteoarthritis). In addition, the GSE157322 dataset comprised 20 patients, including 10 individuals with low BMD (European descent, undergoing total joint replacement surgeries for osteoarthritis) and 10 individuals with high BMD (Polynesian descent, undergoing total joint replacement surgeries for osteoarthritis), considering that previous research has established that women of Polynesian ancestry typically exhibit higher BMD compared to their European counterparts [24].

### Identification of ac4C-modified genes

Recent research has demonstrated that *NAT10* acetylates mRNA, thereby providing significant insights into the epitranscriptome. Specifically, ac4C RNA immunoprecipitation sequencing (ac4C-RIPseq) conducted on both wild-type and *NAT10*-deficient HeLa cells has identified 2,118 genes affected by *NAT10*-mediated ac4C modification. These genes are collectively designated as the ac4C-modified genes [9].

### Screening differentially expressed genes and functional enrichment analysis

The datasets GSE156508 and GSE157322 underwent batch effect correction utilizing the ‘sva’ package in R software [25]. The Limma package (version 3.40.2) within the R software environment was utilized to investigate the differential expression of mRNA. The criteria for identifying differential mRNA expression were established as a P-value of less than 0.05 and a fold change (FC) greater than 1.3 or less than −1.3, a criterion commonly used in exploratory bioinformatics analyses to capture genes with moderate but potentially biologically relevant expression changes [26–28]. Moreover, to further elucidate the potential functions of the identified targets, we conducted a functional enrichment analysis. Gene Ontology (GO) serves as a widely recognized framework for gene function annotation, particularly in the domains of Molecular Function (MF), Biological Process (BP), and Cellular Component (CC). Additionally, Kyoto Encyclopedia of Genes and Genomes (KEGG) enrichment analysis provides a robust tool for examining gene functions and associated high-level genomic functional information. To enhance our understanding of the functional roles of the target genes, we employed the ClusterProfiler package in R to perform GO functional analysis on the potential mRNAs and to enrich the KEGG pathways. In addition, Gene set enrichment analysis (GSEA) was performed based on the log_2_(FC) using GSEA software (http://software.broadinstitute.org/gsea).

### Identification of hub genes and construction of the protein-protein interaction network

The Protein-Protein Interaction (PPI) networks were constructed with the STRING platform (http://cn.string-db.org). Furthermore, the PPI network diagram was plotted by https://www.bioinformatics.com.cn (last accessed on 10 Dec 2024), an online platform for data analysis and visualization. In addition, CytoHubba was used to select hub genes of the PPI network with the Maximal Clique Centrality (MCC) method.

### Analysis of gene expression correlations

The ggstatsplot package in R was employed to generate correlation plots for two genes, while the pheatmap package was utilized to visualize correlations among multiple genes. Spearman’s correlation analysis was conducted to assess the relationships between quantitative variables that do not adhere to a normal distribution. A P-value of less than 0.05 was deemed statistically significant.

### Molecular typing based on ac4C-modified genes

Consensus analysis was conducted on ac4C-modified genes utilizing the R package ConsensusClusterPlus (version 1.54.0). The maximum number of clusters was predetermined at six, with 100 iterations performed by sampling 80 % of the total dataset. Clustering parameters were specified with clusterAlg set to “hc” and innerLinkage configured as “ward.D2”. Subsequently, cluster heatmap analysis was executed using the R package pheatmap (version 1.0.12). In the gene expression heatmap, genes exhibiting a variance below 0.1 were excluded from the analysis. For instances where the number of input target genes exceeded 1,000, the genes were ranked in descending order based on variance, and the top 25 % with the highest variance were selected for visualization.

### Construction of machine learning model for screening crucial ac4C-modified genes

To identify key ac4C-modified genes, three machine learning algorithms—least absolute shrinkage and selection operator (LASSO) logistic regression [29], support vector machine (SVM) [30], and random forest (RF)—were employed using ac4C-modified genes from the combined datasets. Ultimately, the genes identified at the intersection of those selected by LASSO, SVM, and random forest were utilized for diagnosing low BMD.

### Constructing the nomogram and the risk prediction model

To evaluate the diagnostic efficacy of key ac4C-modified genes associated with low BMD, a logistic regression analysis on these genes was conducted within the combined datasets and developed a corresponding logistic regression model. Utilizing the results from this analysis, we employed the ‘rms’ package to construct a nomogram [31]. The receiver operating characteristic (ROC) curve and calibration curve was used to assess the performance of the nomogram, and decision curve analysis (DCA) was performed to evaluate the accuracy and resolution of the logistic regression model by the ‘rmda’ package [32].

### Cell lines and culture condition

The MC3T3-E1 mouse cell line was obtained from the National Infrastructure of Cell Line Resource and subsequently cultured in α-MEM complete medium (Procell, PM150421B) under conditions at 37 °C and 5 % CO_2_.

### Lentivirus construction and infection

Initially, short hairpin RNA (shRNA) lentiviral constructs targeting mouse NAT10 were developed and procured from Genechem (Genechem, China). Subsequently, lentiviruses, along with polybrene at a concentration of 5 μg/mL, were introduced into the culture medium and incubated with MC3T3-E1 cells for 24 hours at a multiplicity of infection (MOI) of 50. Following this incubation period, the medium was replaced. The cells that were successfully infected were then utilized for subsequent analyses. To ensure the selection of transfected cells, puromycin was added to the culture medium at a concentration of 5 μg/mL (MCE, HY-K1057).

### CCK-8 assay

Cell viability was assessed utilizing the CCK-8 assay. Specifically, MC3T3-E1 cells, which had been transduced with lentiviruses (sh-NC and sh-NAT10), were seeded into 96-well plates at a density of 3000 cells per well and measured at 0, 24, 48, 72h post-seeding. The sh-NC group functioned as the control group. Subsequently, 10 μL of the CCK-8 reagent (APE×Bio, K1018) was added to each well, followed by incubation at 37 °C for 1 hour. Absorbance was then measured at 450 nm using a microplate reader (Bio-Rad, USA).

### Alkaline Phosphatase (ALP) Staining

MC3T3-E1 cells were maintained in an osteoblast differentiation medium (OriCell, HUXMX-90021), with medium replacement occurring every three days. On the 14th day, the cells were fixed using 4 % paraformaldehyde (PFA). Post-fixation, ALP staining was performed utilizing a BCIP/NBT ALP kit (Beyotime, C3206), following the manufacturer’s instructions.

### Alizarin Red S (ARS) Staining

MC3T3-E1 cells were maintained in an osteoblast differentiation medium (OriCell, HUXMX-90021), with the medium being refreshed every three days. On the 14th day, the cells were fixed using 4 % PFA. Post-fixation, the cells underwent ARS staining (OriCell, ALIR-1001) for 15 minutes.

### Quantitative real-time PCR

Total RNA was extracted utilizing the TRIzol kit in accordance with the manufacturer’s instructions. The concentration and purity of the RNA were assessed using the NanoDrop 2000 spectrophotometer (NanoDrop, Wilmington, DE, USA). Subsequent experiments were conducted on the cells following the quantification of target mRNA expression levels of *NAT10*, *FADS1*, *NT5DC2*, and *PTGFRN* via quantitative reverse transcription polymerase chain reaction (qRT-PCR). A total of 2 μg of RNA was reverse-transcribed into complementary DNA (cDNA) using the SweScript All-in-One-First-Strand RT SuperMix for qPCR (Servicebio, G3337), as per the manufacturer’s instructions. PCR reactions were conducted using the 2×Universal Blue SYBR Green qPCR Master Mix (Servicebio, G3326). The thermal cycling protocol consisted of an initial denaturation at 95 °C for 30 seconds, followed by 40 cycles of 95 °C for 15 seconds and 60 °C for 30 seconds. A melt curve analysis was performed post-amplification to verify the specificity of the PCR products. Additionally, the housekeeping gene GAPDH served as an endogenous reference for normalization. Relative gene expression levels were calculated using the comparative 2^(−ΔΔCt) method. The specific primer sequences (5′ to 3′) employed were as follows: for *NAT10*, the sense forward primer was TCCAAGGCAACTGTGAAGGC and the reverse primer was GCAGTAGCGAATGTTTGTGGC; for *FADS1*, the forward primer was GAACCCACCA AGAATAAAGCG and the reverse primer was CAGCAGGATGTGAAG CAGGTAG; for *NT5DC2*, the forward primer was GCGCACTTATGGGCTCGTT and the reverse primer was ATACTGGGCCAGCGTGTAGT; for *PTGFRN*, the forward primer was CGTGGATA CCCGCTCTTACC and the reverse primer was GTTCT AGCCAGGTCACGCTT; for *GAPDH*, the forward primer was CCTCGTCCCGTAGACAAAATG and the reverse primer was TGAGGTCAATGAAGGGGTCGT.

### Western blot (WB) analysis

Total protein was extracted from cells or tissues utilizing a RIPA buffer supplemented with PMSF and a phosphatase inhibitor at a ratio of 100:1:1, respectively. The protein concentration was quantified using the BCA Protein Assay Kit (Solarbio, PC0020) and subsequently analyzed via SDS-PAGE. The antibodies employed in this study included NAT10 (Abcam, ab194297, 1:1000 dilution) and GAPDH (Proteintech, 10494-1-AP, 1:10000 dilution).

### Statistical analysis

The results are presented as mean ± standard deviation (SD) derived from a minimum of three independent experiments. Statistical analyses were conducted utilizing R software (Version 4.4.0, available at https://www.rproject.org/). For comparisons between two groups, the Student’s t-test was employed. A P-value of less than 0.05 was considered to indicate statistical significance.

## Results

### Identification of ac4C-modified genes between low BMD group and high BMD group

**Fig. 1:**
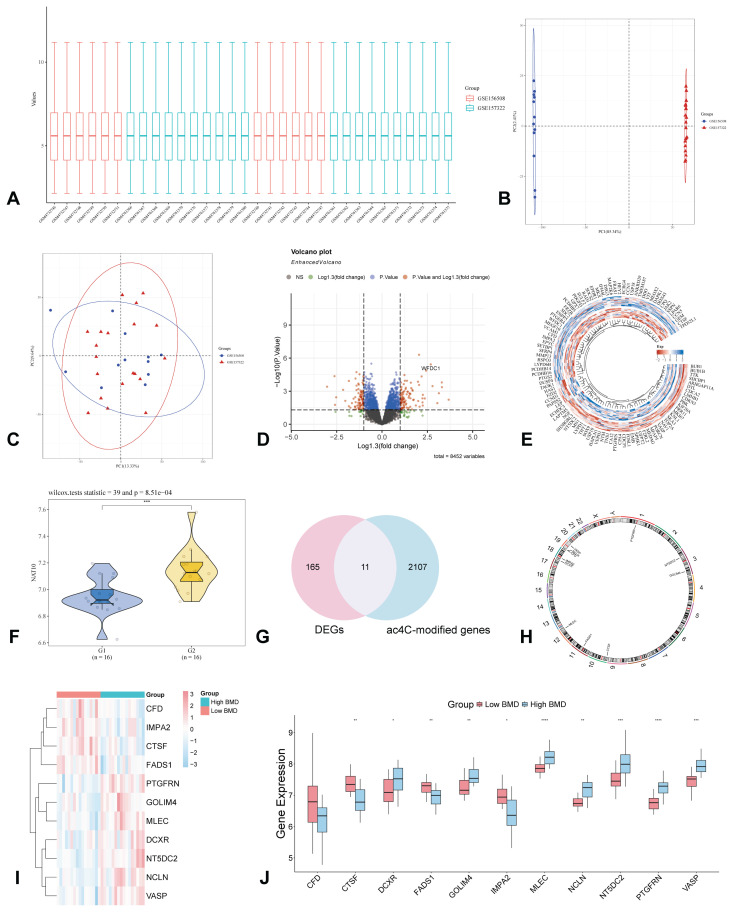
Analysis of differentially expressed genes related to low BMD. (**A**) The boxplot of the primary osteoblast datasets after removing the batch effect; (**B**) PCA of the primary osteoblast datasets before removing the batch effect; (**C**) PCA of the primary osteoblast datasets after removing the batch effect; (**D**) Volcano of differential expression genes in low BMD group vs. high BMD group; (**E**) Heatmap of the expression of differentially expressed genes between low BMD group and high BMD group; (**F**) Violin, dot, and box plot of the expression distribution of the NAT10 gene between low BMD group and high BMD group; (**G**) Venn diagram of intersection of differentially expressed genes and ac4C-modified genes in low BMD group vs. high BMD group; (**H**) Chromosomal map of 11 ac4C-modified genes; (**I**) Heatmap of 11 ac4C-modified genes expression in low BMD group vs. high BMD group; (**J**) Boxplot of differential expression of 11 ac4C-modified genes in low BMD group vs. high BMD group. ****p < 0.0001; ***p < 0.001; **p < 0.01; *p < 0.05. ac4C, N4-acetylcytidine; BMD, bone mineral density; NAT10, N-Acetyltransferase 10.

Initially, datasets GSE156508 and GSE157322, comprising primary osteoblast samples from patients with differing BMD levels (either low or high), were merged to create an integrated GEO dataset, with batch effects systematically removed. The batch effect within this integrated dataset was significantly mitigated through the batch removal process (Fig. 1A). The effectiveness of this batch effect removal was further validated via Principal Component Analysis (PCA), which illustrated a substantial reduction in batch effects among samples originating from various sources within the integrated GEO dataset (Fig.s 1B–1C). Furthermore, the findings indicated a significant reduction in the expression levels of *NAT10* in the low BMD group compared to the high BMD group (Fig. 1F). Additionally, 11 ac4C-modified genes were identified through the intersection of ac4C-modified genes and 176 differentially expressed genes (Fig.s 1D, 1E, 1G, 1I). The chromosomal locations of these 11 ac4C-modified genes are illustrated on the chromosomal map (Fig. 1H). Moreover, all ac4C-modified genes, with the exception of *CFD*, exhibited significant differences between the two groups (*p* < 0.001) (Fig. 1J).

### Functional enrichment analysis and PPI network between low BMD group and high BMD gGroup

**Fig. 2:**
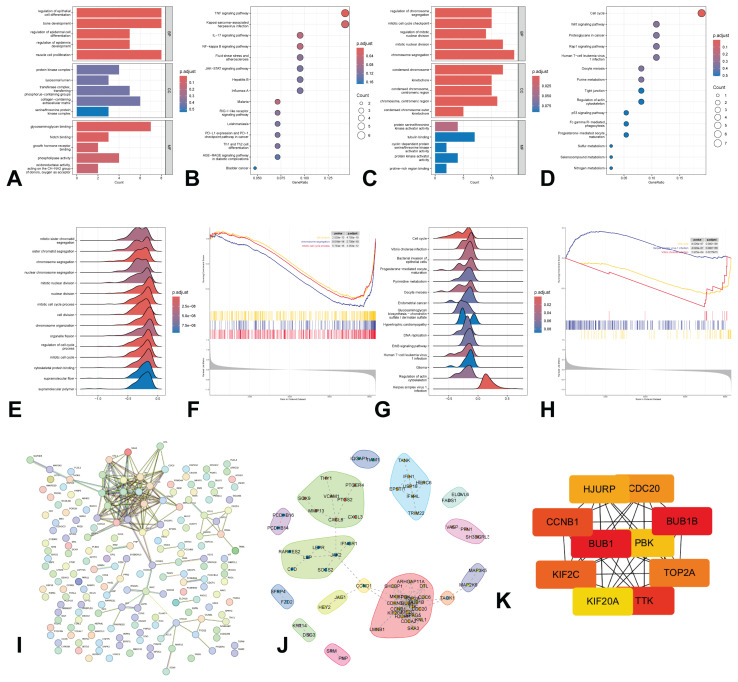
Enrichment analysis of the DEGs and PPI networks. (**A**) GO enrichment of upregulated genes; (**B**) KEGG enrichment of upregulated genes; (**C**) GO enrichment of downregulated genes; (**D**) KEGG enrichment of downregulated genes; (**E–F**) GSEA-GO analysis; (**G–H**) GSEA-KEGG analysis; (**I**) PPI networks; (**J**) Regulatory network among the differentially expressed protein-coding genes; (**K**) Ten hub proteins identification based on MCC values. DEGs, differentially expressed genes; GSEA, Gene Set Enrichment Analysis; GO, Gene Ontology; KEGG, Kyoto Encyclopedia of Genes and Genomes; PPI, Protein-Protein Interaction; MCC, Maximal Clique Centrality.

Using the 176 differentially expressed genes, analyses of GO and the KEGG were performed, as depicted in Fig.s 2A–2D. The analysis identified that 95 upregulated genes were primarily associated with the regulation of epithelial cell differentiation in BP, the protein kinase complex in CC, glycosaminoglycan binding in MF, and the TNF signaling pathway according to KEGG (Fig.s 2A–2B). Furthermore, the analysis showed that 81 downregulated genes were mainly related to the regulation of chromosome segregation in BP, the condensed chromosome in CC, protein serine/threonine kinase activator activity in MF, and the cell cycle pathway in KEGG (Fig.s 2C–2D). Additionally, gene expression in patients with high and low BMD was further analyzed for functional enrichment using GSEA, as shown in Fig.s 2E–2H. The GSEA results indicated significant differences between the low BMD and high BMD groups (Fig.s 2E–2H). Moreover, a protein-protein interaction (PPI) network is presented in Fig.s 2I–2J. Following the analysis using the MCC method, ten genes (*BUB1, BUB1B, CCNB1, CDC20, HJURP, KIF20A, KIF2C, PBK, TOP2A, TTK*) were selected from the PPI network (Fig. 2K).

### Analysis of gene expression correlations

**Fig. 3:**
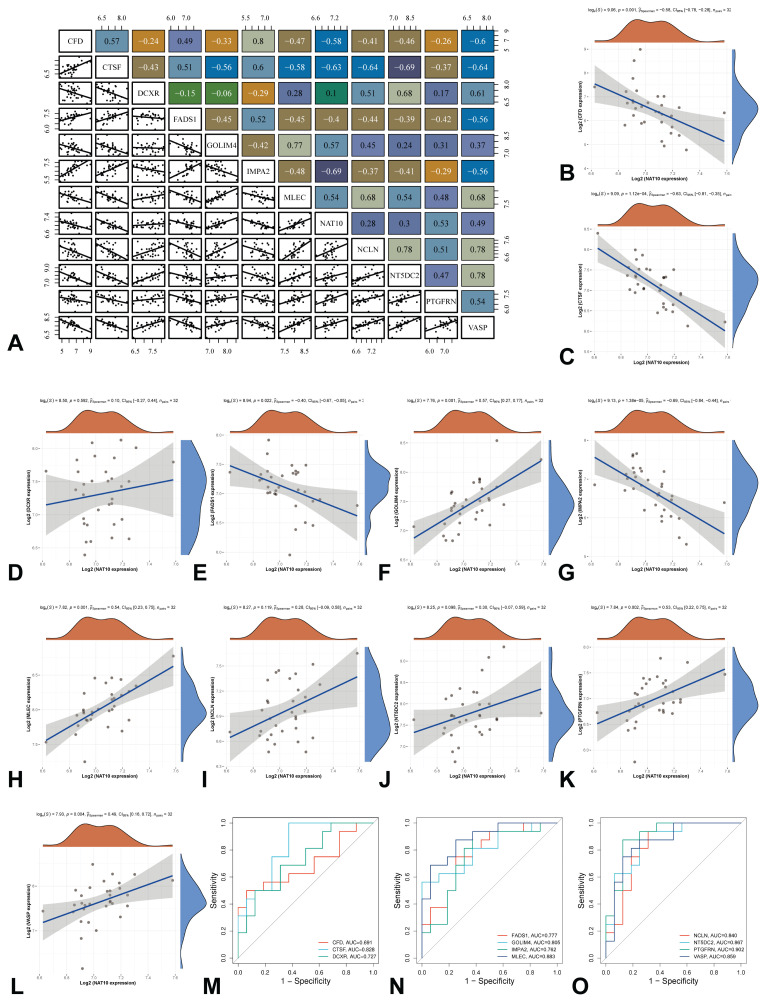
Correlation analysis between NAT10 and ac4C-modified genes. (**A**) Multi-gene correlation heat map; (**B–L**) Correlation between NAT10 and 11 ac4C-modified genes; (**M–O**) ROC curve of the 11 ac4C-modified genes. ac4C, N4-acetylcytidine; NAT10, N-Acetyltransferase 10; ROC, receiver operating characteristic.

Further analysis of ac4C-modified genes across different BMD groups revealed multiple correlations among these genes (Fig. 3A). Specifically, the correlation results between *NAT10* and 11 ac4C-modified genes (Fig.s 3B–3L) indicated that the strongest synergistic effect was observed between *NAT1*0 and *GOLIM4* (Fig. 3F), followed by *NAT10* and *MLEC* (Fig. 3H), and *NAT10* and *PTGFRN* (Fig. 3K). Conversely, the most pronounced competitive effect was identified between NAT10 and *IMPA2* (Fig. 3G), followed by NAT10 and *CTSF* (Fig. 3C), and NAT10 and *CFD* (Fig. 3B). Additionally, ROC curve analysis demonstrated that four genes (*MLEC*, *NT5DC2*, *PTGFRN*, *VASP*) showed high diagnostic accuracy (AUC >0.85), with *PTGFRN* being the strongest predictor (AUC=0.902) (Fig.s 3M–3O).

### Molecular typing based on ac4C-modified genes

**Fig. 4:**
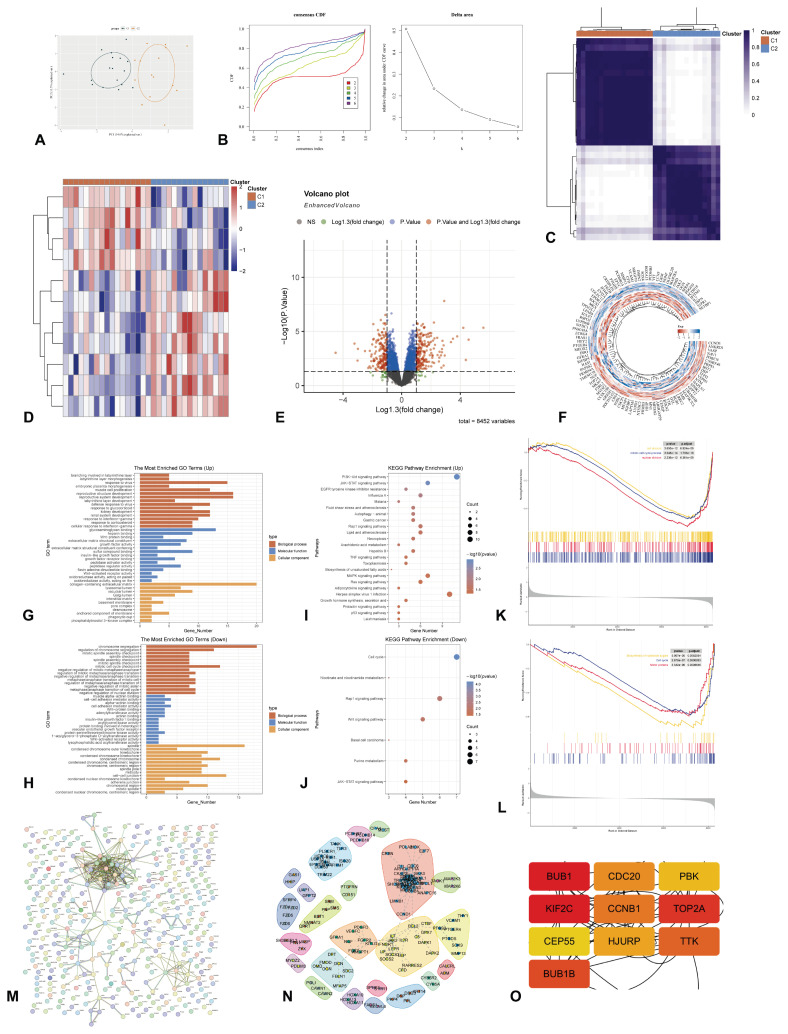
Characteristics based on 11 ac4C-modified genes related molecular typing. (**A**) PCA of different ac4C-modified genes related subtypes; (**B**) Consensus CDF and Delta region; (**C**) Consistent matrix at k=2. The rows and columns of the matrix represent samples; (**D**) Heatmap of differential gene expression between different ac4C-modified genes related subtypes; (**E**) Volcano of differential expression genes between different ac4C-modified genes related subtypes; (**F**) Heatmap of the expression of top 50 differentially expressed genes between different ac4C-modified genes related subtypes; (**G**) GO enrichment of upregulated genes; (**H**) GO enrichment of downregulated genes; (**I**) KEGG enrichment of upregulated genes; (**J**) KEGG enrichment of downregulated genes; (**K**) GSEA-GO analysis; (**L**) GSEA-KEGG analysis; (**M**) PPI networks; (**N**) Regulatory network among the differentially expressed protein-coding genes; (**O**) Ten hub proteins identification based on MCC values. ac4C, N4-acetylcytidine; CDF, cumulative distribution function; GSEA, Gene Set Enrichment Analysis; GO, Gene Ontology; KEGG, Kyoto Encyclopedia of Genes and Genomes; PCA, principal component analysis; PPI, Protein-Protein Interaction; MCC, Maximal Clique Centrality.

To examine the biological distinctions among various subtypes based on ac4C-modified genes, we employed the ConsensusClusterPlus software package to delineate subtypes according to the expression profiles of 11 ac4C-modified genes. At k=2, the classification proved to be both reliable and stable, resulting in the division of samples into cluster1 and cluster2. PCA further validated the significant differences between cluster1 and cluster2 (Fig. 4A–4C). Consensus clustering revealed 358 DEGs between subtypes (197 upregulated, 161 downregulated; Fig 4D–4F), suggesting distinct transcriptional programs. Subsequent analyses of GO and the KEGG pathways were conducted using these 358 DEGs, as illustrated in Fig.s 4G–4J. Additionally, GSEA results demonstrated significant differences between the subtypes associated with different ac4C-modified genes (Fig.s 4K–4L). Furthermore, a PPI network is depicted in Fig.s 4M–4N. Through analysis using the MCC method, ten genes *(BUB1, BUB1B, CCNB1, CDC20, CEP55, HJURP, KIF2C, PBK, TOP2A, TTK*) were selected from the PPI network (Fig. 4O).

### Screening crucial ac4C-modified genes and constructing the risk prediction model

**Fig. 5:**
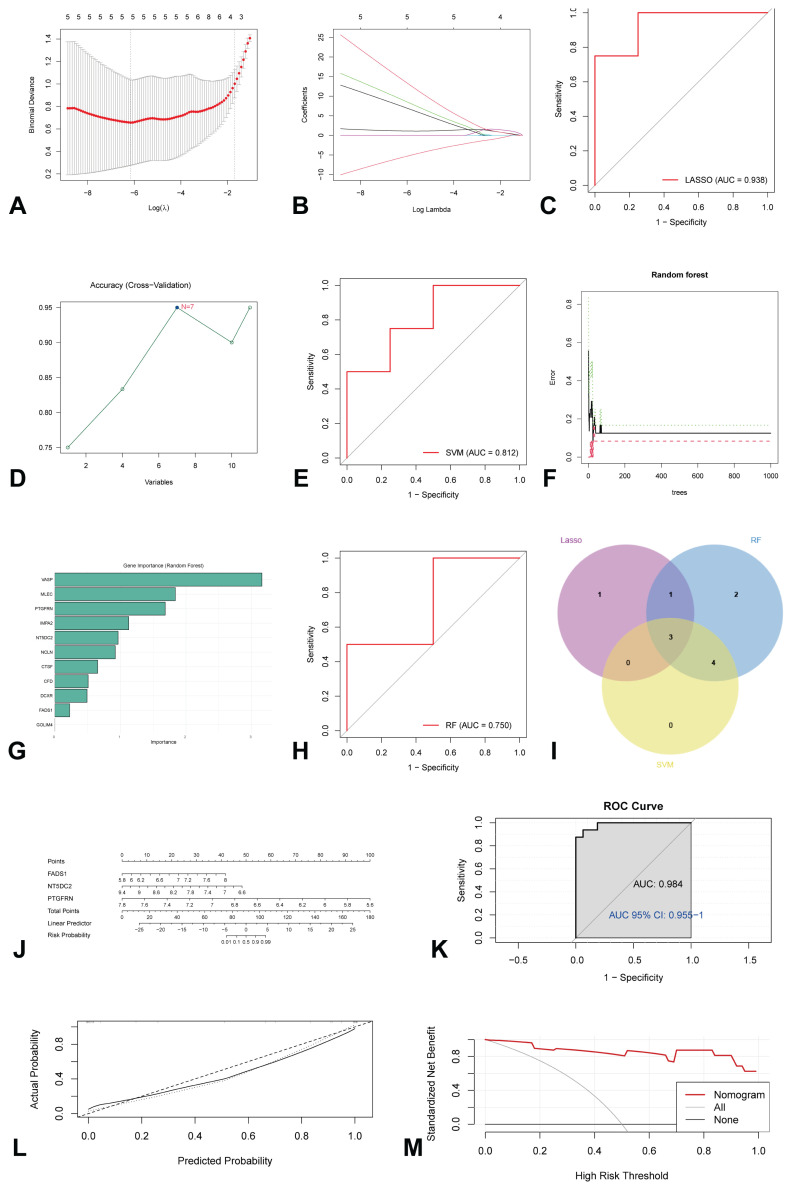
Machine learning algorithm for crucial ac4C-modified genes. (**A**) LASSO plot showed the variations in the size of coefficients for parameters shrank as the value of the k penalty increased; (**B**) Penalty plot of the LASSO model with error bars denoting standard errors; (**C**) ROC curve of the LASSO model; (**D**) Variable screening plot of SVM model; (**E**) ROC curve of the SVM model; (**F**) The error rate confidence intervals for the RF model; (**G**) The importance of genes in RF model; (**H**) ROC curve of the RF model; (**I**) The interaction of the LASSO, SVM, and RF algorithms; (**J**) Nomogram showing the predicted risk for low BMD based on crucial ac4C modified genes; (**K**) ROC curve of discrimination of the nomogram; (**L**) Calibration plot for calibration of the nomogram; (**M**) DCA showing the clinical benefits of the nomogram. ac4C, N4-acetylcytidine; BMD, bone mineral density; DCA, decision curve analysis; LASSO, least absolute shrinkage and selection operator; RF, random forest; ROC, receiver operating characteristic; SVM, support vector machine.

This study further refined the screening process for identifying key ac4C-modified genes by employing three machine learning algorithms: LASSO regression, SVM, and RF. LASSO regression identified a total of five genes (Fig.s 5A–5B), achieving a ROC curve with an area under the curve (AUC) of 0.938 (Fig. 5C). The SVM method demonstrated optimal accuracy when the number of variables was set to seven (Fig. 5D), with an ROC AUC of 0.812 (Fig. 5E). The RF algorithm was utilized to select the top ten genes based on variable importance (Fig.s 5F–5G), resulting in an ROC AUC of 0.750 (Fig. 5H). By integrating the results from the three algorithms, three diagnostic signatures, namely *FADS1*, *NT5DC2*, and *PTGFRN*, were identified as critical ac4C-modified genes (Fig. 5I). Furthermore, a predictive tool for low BMD, specifically a nomogram, was developed by incorporating three ac4C-modified genes: *FADS1*, *NT5DC2*, and *PTGFRN*. Within this nomogram, each significant variable is assigned a score, and the cumulative score of all variables provides a total score indicative of the risk for low BMD (Fig. 5J). The accuracy of the nomogram in diagnosing low BMD was validated through the results of the ROC curve and calibration curve analyses (Fig.s 5K–5L). DCA further demonstrated that the implementation of the nomogram offers clinical benefits to patients with low BMD (Fig. 5M).

### Experimental validation in vitro

**Fig. 6:**
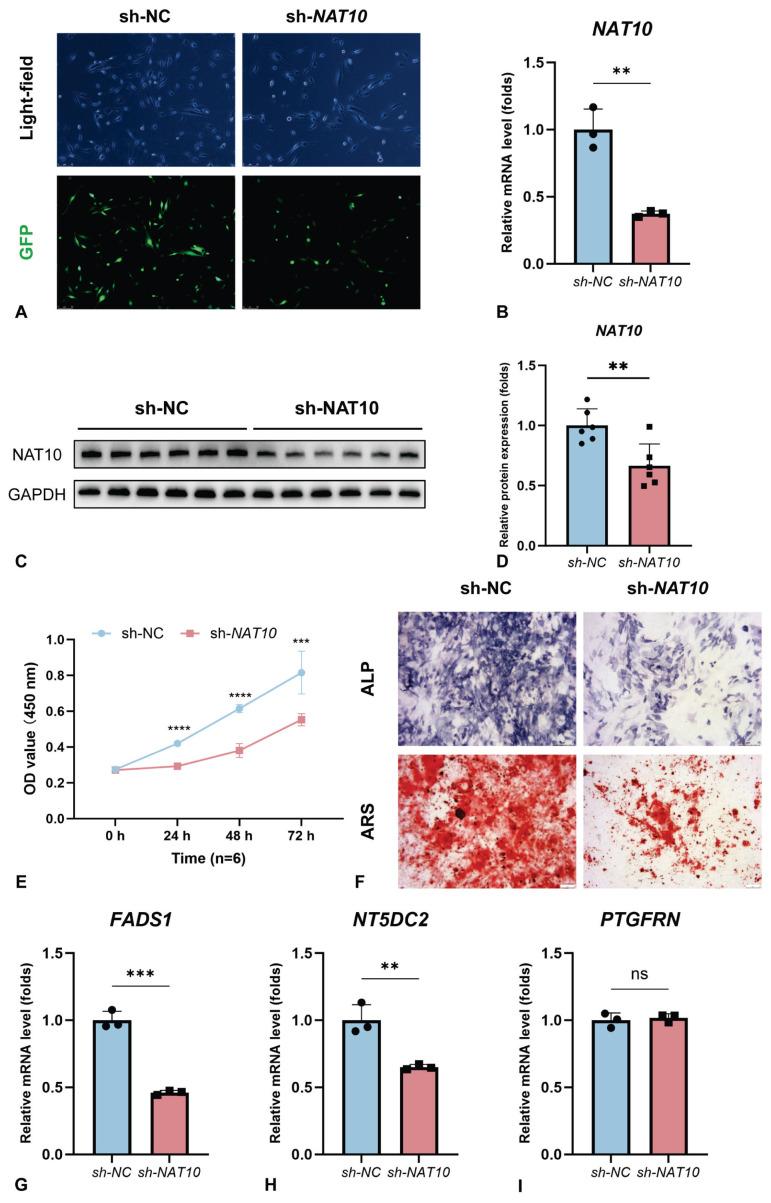
Experimental validation *in vitro*. (**A**) GFP expression after the infection of NAT10 shRNA lentivirus; (**B**) Expression of relative NAT10 mRNA level by qRT-PCR after the infection of NAT10 shRNA lentivirus; (**C–D**) Expression of relative NAT10 protein level by WB after the infection of NAT10 shRNA lentivirus; (**E**) Cell proliferation activity of MC3T3-E1 cells following NAT10 knockdown by CCK-8 assay; (**F**) ALP activity and ARS staining of MC3T3-E1 cells following NAT10 knockdown. (**G–I**) Expression of FADS1, NT5DC2, and PTGFRN of MC3T3-E1 cells following NAT10 knockdown by qRT-PCR. ****p < 0.0001; ***p < 0.001; **p < 0.01. ac4C, N4-acetylcytidine; ALP, alkaline phosphatase; ARS, alizarin red S; CCK-8, Cell Counting Kit-8; FADS1, Fatty Acid Desaturase 1; NAT10, N-Acetyltransferase 10; NT5DC2, 5′-Nucleotidase Domain Containing 2; PTGFRN, Prostaglandin F2 Receptor Inhibitor; qRT-PCR, quantitative real-time polymerase chain reaction; WB, western blot.

Initially, the results from the fluorescence experiment, qRT-PCR, and WB analysis (Fig.s 6A–6D) confirmed the successful establishment of an *NAT10*-knockdown MC3T3-E1 cell model. Specifically, both mRNA and protein expression levels of *NAT10* were markedly diminished in the sh-*NAT10* group relative to the sh-NC group. Additionally, the CCK-8 assay results (Fig. 6E) suggested that *NAT10* knockdown substantially suppressed cell proliferation. Moreover, the data from ALP and ARS staining (Fig. 6F) indicated that *NAT10* knockdown significantly inhibited the osteogenic differentiation of MC3T3-E1 cells. Furthermore, qRT-PCR analysis (Fig. 6G–6I) revealed a significant downregulation of *FADS1* and *NT5DC2* expression following *NAT10* knockdown. However, no significant differences were observed in the expression of *PTGFRN* between the sh-NC group and the sh-*NAT10* group (Fig. 6I).

## Discussion

This study establishes *NAT10* as a critical regulator of osteoblast-mediated bone formation through ac4C-dependent RNA epitranscriptomic mechanisms. By integrating transcriptomic profiling of primary osteoblasts from individuals with divergent BMD and functional validation in osteoblastic models, these findings position the *NAT10*/ac4C axis as a promising therapeutic target for ameliorating osteogenic impairment in OP.

This study observed that not all 11 ac4C-modified genes exhibited a positive correlation with *NAT10* expression, which appears to contradict the classical theory regarding the influence of NAT10-mediated ac4C modifications on mRNA stability and translation efficiency. Paradoxically, subsequent in vitro validation demonstrated that two genes, *FADS1* and *NT5DC2*, which were negatively correlated with *NAT10* expression in bioinformatic analyses, were significantly downregulated following *NAT10* knockdown. This apparent contradiction may be reconciled through several mechanistic perspectives. The regulation of gene expression levels is a complex process involving multiple regulatory mechanisms [33,34]. Similarly, the expression of *FADS1* and *NT5DC2* may be regulated by mechanisms independent of the *NAT10*/ac4C axis. For instance, studies have shown that the transcription factors PATZ1 and SP1/SREBP1c compete in regulating the FADS1 gene. PATZ1 downregulates FADS1 expression by binding to the rs174557 site in the first exon of FADS1, while SP1/SREBP1c has an activating effect [35]. Moreover, *FADS1* mRNA has been shown to be regulated by m6A modification in breast cancer [36]. Therefore, the combined regulation and control of multiple signaling pathways within the context of bone loss may be a significant factor contributing to the negative correlation between *NAT10* and *FADS1*, as well as *NT5DC2*.

This study intriguingly identified *FADS1* and *NT5DC2*, primarily expressed in mitochondria [37,38], as potential targets regulated by the NAT10/ac4C axis. Furthermore, *FADS1* and *NT5DC2* have been demonstrated to have significant associations with mitochondrial metabolism [39,40]. For instance, research suggests that the overexpression of *FADS1* can enhance fatty acid synthesis and triglyceride accumulation by inhibiting the AMPK/SREBP1 pathway [41]. Additionally, *NT5DC2* has been implicated in the regulation of dopamine synthesis through its influence on the phosphorylation of tyrosine hydroxylase [42]. Moreover, accumulating evidence indicates that the expression of genes related to mitochondrial metabolism, such as *FADS1*, is intricately linked to osteoblast function. For example, studies have demonstrated that *FADS1* expression is closely associated with genes involved in bone formation, potentially modulating osteoblast activity by influencing lipid metabolism pathways [43,44]. Consequently, we hypothesize that the *NAT10*/ac4C axis may impact osteoblast function by modulating mitochondrial metabolism. Our comprehensive bioinformatic and experimental analyses propose a regulatory mechanism involving the *NAT10*/ac4C axis and mitochondrial metabolism, with a focus on *FADS1* and *NT5DC2*. However, this proposed model requires extensive validation through diverse methodologies, particularly in vivo metabolic tracing and studies utilizing patient-derived osteoblast models, to ascertain its pathophysiological significance in bone disorders.

This study establishes a foundational framework for utilizing RNA epitranscriptomics to address unmet clinical needs in skeletal disorders, with dual implications. Clinically, the NAT10/ac4C-centric gene signature, demonstrating an AUC of 0.984 for BMD classification, presents a novel diagnostic tool for the early stratification of OP risk. Furthermore, the druggability of NAT10 highlights its potential as a significant therapeutic target, suggesting that small-molecule agonists could be repurposed to promote bone formation and serve as a therapeutic intervention for OP. Scientifically, this study systematically examines the role of NAT10-mediated ac4C modification in osteoblast differentiation and bone formation, which is essential for enhancing our understanding of bone metabolism regulation and for the development of novel therapeutic strategies. We anticipate that elucidating the complex interactions between epigenetic modifications and mitochondrial function will remain a productive area for future research.

This study has several limitations. Firstly, MC3T3-E1 is an immortalized cell line, and its ac4C modification profile may differ from that of primary osteoblasts, necessitating the validation of key findings in primary human osteoblasts. Secondly, although changes in target gene expression were validated, direct assessment of ac4C modification levels, such as through acRIP-qPCR, was not conducted. Future research is needed to confirm whether *NAT10* knockdown specifically reduces ac4C modification on the target genes, such as *FADS1*, *NT5DC2*, and *PTGFRN*. In addition, developing targeted *NAT10* agonist drugs and investigating upstream signals regulating *NAT10* activity (such as mechanical loading or hormones) represent important future research directions, which may provide novel therapeutic paradigms and a foundation for enhancing bone formation and treating OP.

In conclusion, the present study found 11 ac4C-modified genes related to different BMD statuses, namely *CFD, CTSF, DCXR, FADS1, GOLIM4, IMPA2, MLEC, NCLN, NT5DC2, PTGFRN,* and *VASP*. Moreover, the prediction model constructed using ac4C-modified genes demonstrated excellent performance in distinguishing different BMD statuses. These findings identify NAT10-mediated ac4C modification as a crucial regulator of osteoblast activity and gene expression programs related to human BMD. This research provides novel insights into the regulation of bone metabolism and suggests potential diagnostic markers and therapeutic targets for OP.
